# Death of Professor Timothy Childs

**Published:** 1865-10

**Authors:** 


### Death of Professor Timothy Childs.

We are pained to announce the death of this well known and highly esteemed member of the profession, at Norwich, Conn., on the 3d inst., from the effects of an excessive dose of morphine, administered by himself. Dr. Childs was a native of Pittsfield, in this State, and filled for many years an honorable place as Professor of Anatomy and Surgery in the Berkshire Medical College, and more recently as Professor of Anatomy in New York Medical College. He also served with distinction as assistant-surgeon to a Massachusetts regiment in the Mexican war, and on numerous occasions acted as a volunteer after the great engagements of the recent war. He was endeared to a large circle of friends by his kindness of heart and genial disposition, and his death will be widely mourned. It is thought he was laboring under temporary insanity at the time of his death.—*Boston Medical Journal.*

